# Impact of endometriosis on oocyte morphology in IVF-ICSI: retrospective study of a cohort of more than 6000 mature oocytes

**DOI:** 10.1186/s12958-021-00798-x

**Published:** 2021-10-16

**Authors:** Camille Robin, Audrey Uk, Christine Decanter, Hélène Behal, Pierre Collinet, Chrystèle Rubod, Anne-Laure Barbotin, Geoffroy Robin

**Affiliations:** 1 Service de Gynécologie Endocrinienne Et Médecine de La Reproduction, CHU Lille, Assistance Médicale À La Procréation Et Préservation de La Fertilité, Hôpital Jeanne de Flandre, Service dAvenue Eugène Avinée, 59000 Lille, France; 2 Institut de Biologie de La Reproduction-Spermiologie-CECOS, Hôpital Jeanne de Flandre, Centre Hospitalier Et Universitaire, 59000 Lille, France; 3 EA 4308 Gamètogenèse Et Qualité du Gamète, Faculté de Médecine Et CHU de Lille, F-59000 Lille, France; 4 EA 2694, Santé Publique : Épidémiologie Et Qualité Des Soins, Univ. Lille, CHU Lille, Unité de Biostatistiques, F-59000 Lille, France; 5 Service de Chirurgie Gynécologique, Hôpital Jeanne de Flandre, Centre Hospitalier Et Universitaire, 59000 Lille, France; 6 Faculté de Médecine, Université de Lille, 59045 Lille, France

**Keywords:** Endometriosis, Oocyte morphology, In vitro fertilization, Oocyte quality, Endometrioma

## Abstract

**Background:**

Infertility associated with endometriosis can be explained by several non-exclusive mechanisms. The oocyte plays a crucial role in determining embryonic competence and this is particularly relevant for in vitro fertilization (IVF) outcomes. According to some authors, the morphology of oocytes could also be a non-invasive marker of oocyte quality. The aim of this study was to evaluate the relationship between endometriosis and oocyte morphology after controlled ovarian stimulation for intracytoplasmic sperm injection (ICSI) on a large oocyte cohort.

**Methods:**

Single-center comparative retrospective study in the academic In Vitro Fertilization (IVF) unit of the Lille University Hospital. A total of 596 women treated for IVF-ICSI with ejaculated spermatozoa for sperm alterations were included. They were classified as endometriosis (n = 175) or control groups (n = 401). The morphological evaluation of 2,016 mature oocytes from 348 cycles of patients with endometriosis was compared with that of 4,073 mature oocytes from 576 control cycles. The main outcome measures were Average Oocyte Quality Index (AOQI) and metaphase II oocyte morphological scoring system (MOMS). Comparison of groups was carried out by a mixed linear model and by a generalized estimation equation model with a "patient" random effect to consider that a patient might have several attempts.

**Results:**

No difference in AOQI and MOMS scores was found between endometriosis and control women (*adjusted p* = 0.084 and 0.053, respectively).

In case of endometriosis, there were significantly fewer metaphase II oocytes retrieved, embryos obtained, grade 1 embryos and number of cumulative clinical pregnancies compared to controls. In the endometriosis group, endometriosis surgery was associated with a reduced number of mature oocytes retrieved, and the presence of endometrioma(s) was associated with some abnormal oocyte shapes. Nevertheless, no difference concerning the AOQI and MOMS scores was found in these subgroups.

**Conclusion:**

Endometriosis does not have a negative impact on oocytes’ morphology in IVF-ICSI.

**Trial registration:**

On December 16, 2019, the Institutional Review Board of the Lille University Hospital gave unrestricted approval for the anonymous use of all patients’ clinical, hormonal and ultrasound records (reference DEC20150715-0002).

**Supplementary Information:**

The online version contains supplementary material available at 10.1186/s12958-021-00798-x.

## Background

Endometriosis is characterized by the presence of endometrial glands or stroma outside the uterine cavity [1, 2]. It would affect about 10–15% of women aged 15–49 years-old and 25–50% of patients managed for infertility [3–5]. The American Fertility Society classification (AFS classification), based on laparoscopic results, is the most widely used to define the severity of the pathology [6]. However, this anatomical classification does not allow describing deep endometriosis correctly [7]. It is also poorly predictive of chances of spontaneous or induced pregnancy and has little correlation with the severity of the symptoms presented by the patients [7]. Many pathophysiological hypotheses for endometriosis exist and clarify the heterogeneity of its localizations [1, 2, 8–10]. Most pathologists suggest a probable combination of the different hypotheses. Endometriosis is thus a multifactorial disease, resulting from the combined action of genetic, hormonal, immunological, angiogenic and environmental factors [1, 2, 9–11].

Infertility associated with endometriosis can be explained by several non-exclusive mechanisms: organic damage to certain organs such as the fallopian tubes, chronic inflammation of the pelvic cavity, which can disrupt gamete survival and fertilization, disruption of the physiological processes of implantation, ovarian abnormalities (alteration of the quantity and/or quality of the oocyte, potential alteration of the oocyte microenvironment in women with endometriosis related to a variation of the cytokine expression profile) [4, 11–15].

The oocyte plays a crucial role in determining embryonic competence and this is particularly relevant for in vitro fertilization (IVF) outcomes [16]. For ethical reasons, oocyte quality has generally been studied indirectly by non-invasive procedures such as the evaluation of the cumulus cells surrounding the oocytes and/or the analysis of follicular fluid [5]. However, it is not certain that these approaches truly reflect the quality and competence of the oocyte [5]. According to some authors, the morphology of oocytes could also be a non-invasive marker of oocyte quality [16–20]. Thus, Van Blerkom and Henry's work [20] hypothesized that oocyte quality may be related to certain morphological irregularities easily identifiable by light microscopy [16, 20]. Oocyte morphology is evaluated by analysis of cumulus cells, nuclear maturation, as well as the appearance of the cytoplasm and extra-cytoplasmic structures [18]. An oocyte with " ideal " morphology should present nuclear maturation criteria and have a normal size, cytoplasm, zona pellucida thickness and perivitelline space [16, 19]. According to some authors, it is the combination of all these morphological evaluations that would make it possible to assess, in part, the oocyte quality [16, 21].

The majority of studies evaluating oocyte quality in endometriosis have mainly focused on the immaturity rate of oocytes collected in IVF (i.e. oocytes that cannot be used in practice) and not on the morphological aspect of mature oocytes that can be used for IVF [5]. Nevertheless, some authors have shown a higher prevalence of oocyte morphological alterations, such as the presence of dark central granulations, an abnormal zona pellucida and/or other intra- and extra-cytoplasmic abnormalities [18, 22, 23]. However, these studies were conducted in small samples, and each of them investigated only a limited number of morphological abnormalities. Several publications seem more supportive of an alteration in oocyte quality rather than a defect in endometrial receptivity in the context of endometriosis [5]. Nevertheless, despite a decrease in the number of mature oocytes retrieved and the number of embryos obtained, the rates of clinical pregnancy and delivery in IVF with or without ICSI do not differ in patients with endometriosis compared with unaffected patients [24–27], except in case of deep pelvic endometriosis lesions as suggested by Ashrafi et al. [28].

We aimed with the current study to investigate whether the morphology of mature oocytes obtained in IVF-ICSI may be influenced by the presence of endometriosis in a large cohort of mature oocytes. The secondary objective of this study was to analyze the influence of the characteristics of endometriosis on oocyte morphology.

## Material and methods

This is a monocentric retrospective study performed in the IVF unit of the University Hospital of Lille (France).

### Study Design and population

Patients were included from the French database JFIV® software (version 1.8; RD Services, Langlade, France) over the period January 2007 to December 2019 using the following criteria: women aged 18 to 42 years who underwent IVF with intracytoplasmic microinjection of ejaculated sperm for sperm alterations.

The following exclusion criteria were applied: patients with polycystic ovary syndrome (according to the recently revised Rotterdam criteria) [29] or any other etiology of anovulation (premature ovarian failure, functional hypothalamic amenorrhea, hyperprolactinemia, other congenital or acquired gonadotropic deficits) or hyperandrogenism (non-classical adrenal hyperplasia, Cushing syndrome), patient with severe or morbid obesity (Body Mass Index (BMI) ≥ 35 kg/m2), karyotype abnormalities in at least one of the two members of the couple, ICSI with surgical sperm in case of azoospermia, patients who underwent complete endometriosis surgery, modified-natural cycle protocol, absence of mature oocytes collected at oocyte retrieval.

The population was divided into two groups according to the notion of a history of endometriosis or not. Control patients had no history of endometriosis. The second group consisted of patients with deep pelvic and/or ovarian endometriosis, operated or not.

As this study was retrospective and without intervention, the opinion of the Ethics Committee on the study was not required. All patients had given prior consent for the use of their clinical, hormonal and ultrasound record. On December 16, 2019, the Institutional Review Board of the Lille University Hospital gave unrestricted approval for the anonymous use of all patients’ clinical, hormonal and ultrasound records (reference DEC20150715-0002).

### Infertility exploration assessment

The infertility assessment included for all patients: a query on the couple's antecedents and lifestyles, calculation of BMI, hormone assays associated with an ultrasound examination for evaluation of ovarian reserve, search for endometriosis lesions and counting of the antral follicles at the beginning of the follicular phase of the menstrual cycle (between D2 and D5 of the cycle). The ultrasound examination was performed with a Voluson E8 Expert ultrasound machine with an endovaginal probe at 5–9 MHz (General Electric Systems, Velizy, France) according to the "real-time two-dimensional (2D)" procedure [30].

### Diagnosis of endometriosis

The diagnosis of endometriosis was made either by endovaginal ultrasound imaging associated with a pelvic Magnetic Resonance Imaging (MRI) performed in our hospital, or surgically with anatomo-pathological confirmation, according to the French National College of Ob-Gyn (CNGOF) guidelines [8]. Deep endometriosis was defined as described by Collinet et al. [8].

The history of endometriosis surgery consisted of any partial endometriosis surgery. All patients had one or more endometriosis lesions at the time of stimulation.

### Biological analysis

The AMH, expressed in pmol/l, was measured prior to any attempt (measurement by the semi-automated 2nd generation AMH-EIA A11893 Immunotech semi-automated enzyme immunoassay kit from Beckman Coulter (Villepinte, France) for samples taken before 01/01/2016, then by the automated Access Dxi B13127 automated assay from Beckman Coulter (Villepinte, France) for samples taken after this date). Due to the different sensitivities of these two techniques, the results were secondarily homogenized using the following conversion formula: AMH-EIA = (AMH Dxi- 0.44) / 0.775) published by Pigny et al. [31]**.** Estradiol (chemiluminescence technique, Architect Axsym® multi-parameter biochemical automaton, Abbott Laboratories (Mandaluyong, Philippines)) was evaluated at each control during controlled ovarian stimulation.

### Procedures for controlled ovarian hyperstimulation (COH)

Patients received either a long agonist or an antagonist protocol. Daily subcutaneous injections of recombinant FSH (Follicle-stimulating Hormone) or HMG (Human Menopausal Gonadotropin) were performed. The starting dose was selected according to age, BMI, Antral Follicle Count (AFC) and AMH. Ovulation was triggered by subcutaneous injection of 250 µg of recombinant hCG (Human Chorionic Gonadotropin) (Ovitrelle®, Merck Serono, Lyon, France) or 0.2 mg of a short half-life GnRH (Gonadotropin Releasing Hormone) agonist (Triptorelin 0.2 mg) if there was a risk of ovarian hyperstimulation syndrome. Thirty-six hours after this injection, trans-vaginal ultrasound-guided oocyte retrieval was performed.

### Oocytes morphological evaluation

The evaluation of maturity and oocyte morphology post-decoronization was carried out approximately 2 h after the retrieval just before sperm injection, at X400 magnification using a Leica DMIRB inverted microscope as previously described [32]. In this study, two oocyte morphology scores were used:

- the AOQI (Average Oocyte Quality Index) score established by Sigala et al. [32] in 2015. This score counts the number of abnormalities in oocyte morphology on 7 items (cytoplasmic granularity, zona pellucida anomaly (irregular shape or thickened zona pellucida), presence of intracytoplasmic vacuoles, material in the perivitelline space, anomaly of the first polar body, large perivitelline space, oocyte shape) and corresponds to the ratio of the total number of abnormalities to the number of metaphase II oocytes collected.

- the MOMS (metaphase II oocyte morphological scoring system) score was calculated for each oocyte. This score published by Rienzi et al. [33] in 2008 attributes a different coefficient to 5 abnormalities depending on their impact on the outcome of the attempts (fertilization rate, rate of zygotes obtained, rate of embryos obtained, pregnancy rates…). The average MOMS scores of the oocytes collected per attempt were then calculated by the ratio of the sum of the MOMS scores of the oocytes to the number of metaphase II oocytes collected on the attempt.

### Embryos morphological evaluation

Sperm microinjection using the ICSI technique was performed on each M2 oocyte. An evaluation of normal diploid fertilization was made at 16—18 h after the injection, by observation of the two pronuclei and the second polar body (PB) expelled in the perivitelline space (PVS). Early cleavage was observed 27 h after injection. Embryo quality was estimated at 44—46 h (or 68 h) from injection. Embryo quality classification in our IVF laboratory is based on the number and size of blastomeres, the degree of fragmentation, and the presence or absence of multi-nucleated blastomeres according to the Istanbul Consensus Conference [17]. On day 2, an embryo was considered to be of good quality if it had 4 blastomeres of equal size, without multi-nucleation and with less than 10% fragmentation. If the embryo transfer was to take place on day 3 after injection, a good quality embryo had to have 8 cells of equal size, without multi-nucleation and with less than 10% fragmentation. Only supernumerary embryos of good quality were frozen for subsequent embryo transfers. Following embryo transfer on day 2 or day 3, all supernumerary embryos (i.e. which were not meet criteria for transfer or freezing) were cultured until day 5 or 6.

### Outcome of the attempts

The transfer of cleaved-staged embryo(s) was performed at D2 or D3 post-oocyte retrieval. The luteal phase support consisted of daily administration of 600 mg vaginal progesterone or 30 mg dydrogesterone per os in 3 doses started on the evening of the oocyte retrieval. A “freeze-all” strategy of the entire embryonic cohort was implemented when the patient was at risk of ovarian hyperstimulation syndrome or when progesterone levels at the end of stimulation were abnormally high. A plasma pregnancy test was routinely performed 14 days after embryo transfer. If a pregnancy was confirmed, luteal phase support was maintained until 8 weeks of pregnancy. The comparison of the two groups was based on fertilization rates (number of zygotes obtained / number of metaphase II oocytes), the number of embryos obtained, the percentage of grade 1 embryos and the cumulative number of clinical pregnancies.

### Statistical analysis

Categorical parameters were expressed as frequencies and percentages. Continuous parameters were expressed as mean and standard deviation or as median and interquartile range. Distribution of continuous parameters were checked graphically and using Shapiro Wilk test.

The comparison of the two groups (Endometriosis vs controls) for the characteristics of the first cycle was performed using a Chi-square test for smoking and using Student t test for continuous parameters.

A linear mixed model was used to compare continuous dependent variables between the two groups, with patient as random effect to take into account for the correlation between the cycles of a same patient. A generalized estimating equation (GEE) model with patient as random effect was used for the others parameters: one GEE model with negative binomial distribution and logit link function (GEE1 model) for the count dependent variables; another one with a binomial distribution and a link logit function (GEE2 model) for the binary dependent variables.

The GEE1 model was performed for the maturation rate, considering the number of MII oocytes as dependent variable and the number of oocytes retrieved as offset; for the fertilization rate, considering the number of zygotes as dependent variable and the number of MII oocytes as offset; for the top embryos rate, considering the number of top embryos as dependent variable and the number of embryos obtained as offset. All offset variables were log-transformed.

A Mann–Whitney U test was used to compare the cumulative implantation rate between two groups. A non-parametric generalized linear model was used to compare the cumulative implantation rate between the endometriosis group vs the control group adjusted on confounding factors.

Analyses were adjusted on the woman’s age, the woman’s smoking status, the body mass index, total dose of FSH, stimulation protocol, stimulation product and the attempt rank (List 1) for the oocyte quality parameters, the fertilization rate, the rate of top embryos, the cumulative pregnancy rate.

Analyses were adjusted on the confounding factors of the List 1 and on the AMH rate for the following parameters: number of follicles over 15 mm, number of oocytes retrieved, number of MII oocytes, the mature oocytes rate relative to the number of oocytes retrieved, the mature oocytes rate relative to the number of follicles over 15 mm, total number of embryos obtained.

For adjusted analyses, missing parameters values were handled by multiple imputation procedures. Missing data were imputed under the missing at random assumption using a regression switching approach (chained equation with m = 10 imputations) with predictive mean matching method for continuous variables and logistic regression (binary, ordinal, or polynomial) for qualitative variables [34]. The imputation procedure were performed using the main baseline characteristics and outcomes, and estimates obtained in the different imputed data sets were combined using the Rubin’s rules [35, 36].

Data were analyzed using the SAS software (SAS Institute Inc, Cary, NC, USA) and all statistical tests were performed with a 2-tailed alpha risk of 0.05.

## Results

A total of 175 patients were included in the endometriosis group and 401 in the control group. They were aged between 19 and 41 years, and had been treated with IVF-ICSI attempts in our IVF unit Thus, 348 ICSI cycles, corresponding to the morphological evaluation of 2016 metaphase II oocytes, in the endometriosis group and 576 cycles, representing 4073 mature oocytes, in the control group were analyzed.

Table 1 shows the clinical characteristics of the patients at the time of their first IVF-ICSI attempt.

**Table 1:**
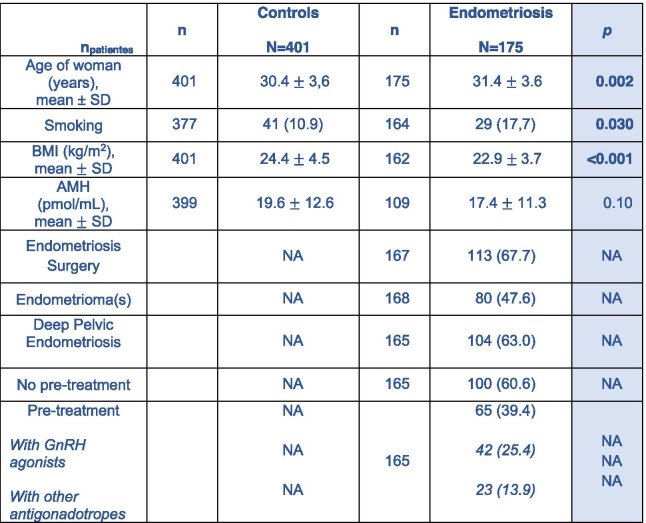
Clinical Characteristics of Patients

Comparisons of ICSI cycles, embryo quality, and outcome of attempts in control and endometriosis patients are presented in Table 2. The number of cycles that required a freeze-all strategy did not differ between the endometriosis and control groups. A total of 117 cycles did not have embryo transfer, of which 49 were in patients with endometriosis. The stimulation methods differed significantly between the 2 groups in terms of protocol type and gonadotropins used, with more stimulation by agonist protocol and HMG in the endometriosis group. Stimulation duration, total FSH dose, and estradiol level at the triggering day were significantly higher in the endometriosis group compared to the control group. The number of follicles over 15 mm on the day of triggering, total number of retrieved oocytes, number of retrieved metaphase II oocytes, and mature oocytes / number of follicles over 15 mm on ultrasound ratio were significantly lower in the endometriosis group, even after adjustment. In the endometriosis group, the total number of embryos obtained, the rate of grade 1 embryos, the cumulative clinical pregnancy rate and the cumulative implantation rate were significantly lower than in Controls. These differences remained after adjustment.

**Table 2:**
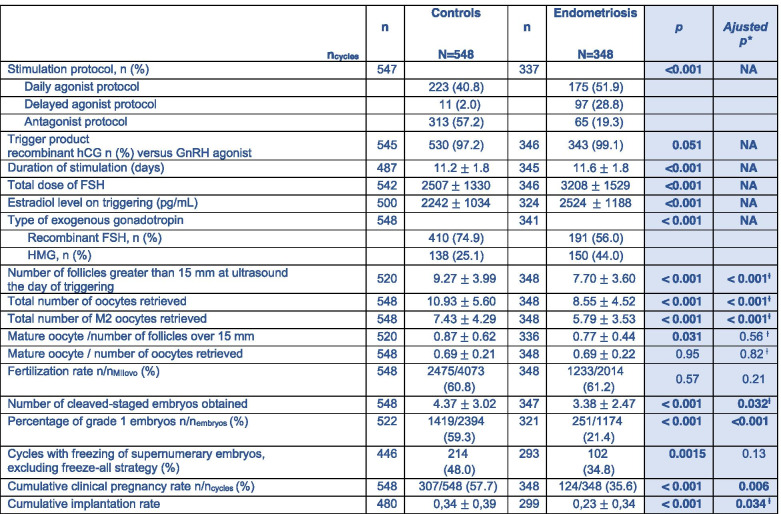
Characteristics of ICSI cycles and comparison of embryo quality and outcome of attempts between control and endometriosis patients

Values are presented by mean and standard deviation unless otherwise stated. * adjustment on: age, tobacco, BMI, rank of attempt, stimulation protocol, stimulation product, total dose of FSH administered after 10 imputations to process missing data. Ɨ adjustment on AMH in addition hCG: Human Chorionic Gonadotropin ; GnRH: Gonadotropin Releasing Hormone ; FSH: Follicle-Stimulating Hormone ; HMG: Human Menopausal Gonadotropin ; BMI: Body Mass AMH: Antimullerian Hormone ; FSH: Follicle Stimulating Hormone ; M2: Metaphase II ; NA : Not Applicable.

Comparison of oocyte morphology according to the history of endometriosis is shown in Table 3. The two groups differed significantly before and after adjustment on the criteria: fragmented first polar body, abnormal oocyte shape, and presence of intracytoplasmic vacuoles. The "presence of perivitelline material" anomaly was significantly greater in the control group, but not after adjustment. No difference on AOQI and MOMS scores was found.

**Table 3:**
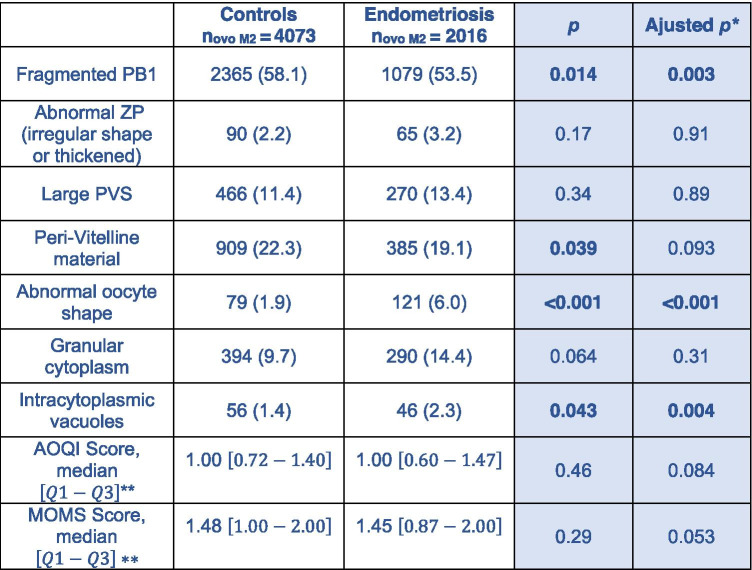
Comparison of oocyte morphology

Values are presented by number of oocytes and percentage, unless otherwise stated. No missing data for these parameters. * adjustment for: age, smoking, BMI, AMH, rank of attempt, stimulation protocol, stimulation product, total dose of FSH administered ** the values are calculated at cycle level PB1: First Polar Body ; ZP: Zona Pellucida ; PVS: Péri-Vitelline Space ; AOQI : Average Oocyte Quality Index ; MOMS: metaphase II oocyte morphological scoring system ; BMI: Body Mass Index ; AMH: Antimullerian Hormone ; FSH: Follicle Stimulating Hormone

Table 4 shows comparison of oocyte morphology and cycle outcomes according to disease characteristics and COH in the endometriosis group. The history of endometriosis surgery impacted the results with regard to the number of oocytes retrieved and the number of mature oocytes collected. The absence of pre-stimulation treatment was associated with a significantly higher number of mature oocytes retrieved. Similarly, recombinant FSH stimulation resulted in a statistically higher number of total oocytes and metaphase II oocytes being obtained during oocyte retrieval compared to HMG stimulation.

**Table 4:**
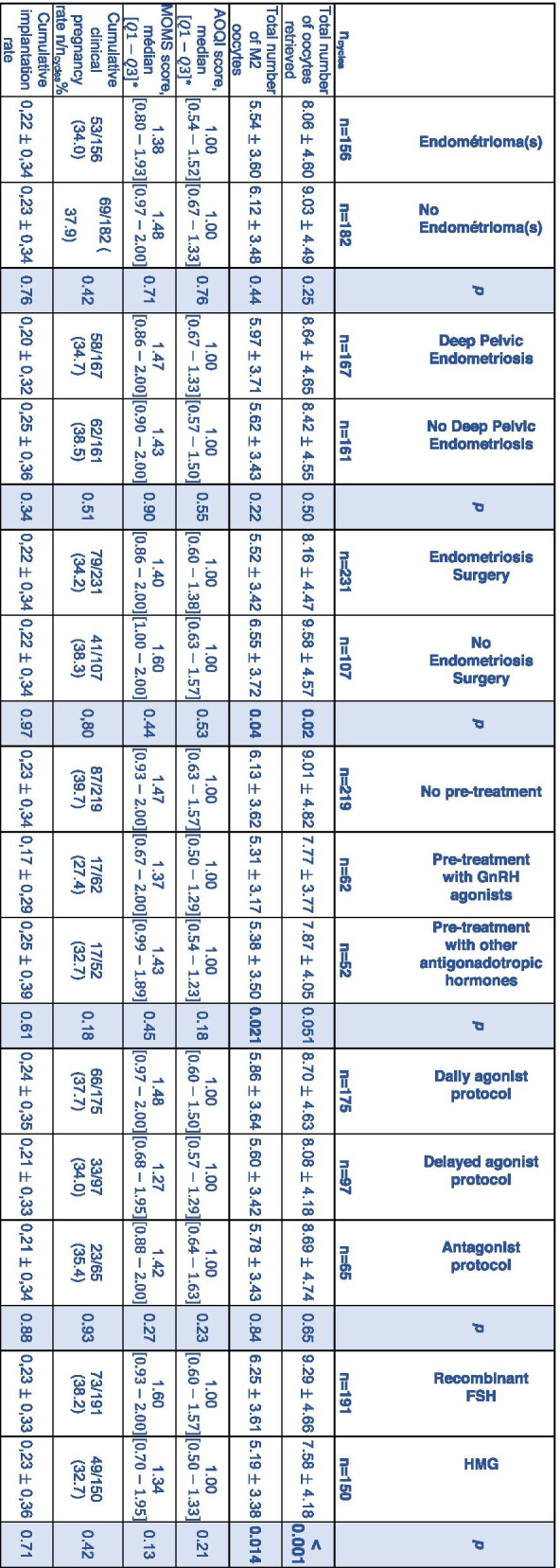
Comparison of oocyte morphology and cycle outcomes according to disease and stimulation characteristics in the endometriosis group

Values are presented by mean and standard deviation unless otherwise stated. * Values are calculated at cycle level AOQI: Average Oocyte Quality Index ; MOMS: metaphase II oocyte morphological scoring system ; M2: Metaphase II ; GnRH: Gonadotropin Releasing Hormone ; FSH: Follicle-Stimulating Hormone ; HMG: Human Menopausal Gonadotropin

Details of the morphological oocyte abnormalities according to the characteristics of endometriosis are presented in the supplemental table. Only the presence of endometrioma(s) was significantly associated with oocyte shape abnormalities.

## Discussion

To our knowledge, this is the first study comparing AOQI and MOMS oocyte morphological evaluation scores in endometriosis compared to controls in IVF-ICSI.

According to our study, endometriosis does not seem to have a significant impact on oocyte morphology in IVF-ICSI. Although the oocytes from the endometriosis group were more frequently misshapen and had intracytoplasmic vacuoles more often than those from the controls, the first polar body was less often fragmented in this group. In addition, the AOQI and MOMS scores, known to assess oocyte morphology, did not differ between the two groups [32, 33]. These results disagree with those previously published. Indeed, Kasapoglu et al. [37], in a retrospective analysis of 1,568 mature oocytes, found a significant increase in morphological abnormalities of the cytoplasm, the zona pellucida and the first polar body in patients with endometriosis compared to controls. Similarly, Shebl et al. [38], in a cohort of 2,343 mature oocytes, have observed a lower number of oocytes with normal morphology in their endometriosis group. In the current study, we preferred to consider all the oocyte abnormalities listed in the MOMS and AOQI scores and not focus on only some of them.

The impact of oocyte morphology on the results of IVF-ICSI is still debated [18]. The meta-analysis of Setti et al. [39] claimed that fertilization rates are significantly associated with the absence of 4 oocyte morphological abnormalities (first polar body and perivitelline space enlarged, refractive bodies and intracytoplasmic vacuoles). These hypotheses are supported by other publications [18, 33, 40–43]. Regarding the aspect of the first polar body, some publications argued that its shape may have more impact on IVF outcomes than its fragmentation [18, 22, 41, 43]. For other authors, the thickness of the zona pellucida also seems to affect the potential for embryonic development [44, 45]. Conversely, other experts assert that the morphology of the first polar body, the size of the perivitelline space and the appearance of the zona pellucida are only phenotypic variations that are more likely to be due to in vitro culture conditions and/or female age, without any real impact on IVF results [16–18]. Furthermore, Ten et al. [46] described increased chances of obtaining good quality embryos in case of a larger perivitelline space. The retrospective study of Chamayou et al. [47] and the prospective analysis of Ashrafi et al. [48] concluded that oocyte dysmorphisms probably have an impact on fertilization rates, but not on implantation or pregnancy rates in IVF-ICSI. Thus, morphological study of the oocyte provides only partial information on the potential for fertilization and embryonic development.

Apart from the analysis of oocyte morphology, other more invasive procedures have been proposed to evaluate oocyte quality: meiotic spindle analysis, oocyte mitochondrial capital assessment, cytogenetic and molecular analysis of first polar body [5, 49–52]. Their relevance is still controversial. Nevertheless, in case of endometriosis, some studies have shown an increased risk of chromosomal misalignment leading to increased rates of aneuploidy [5, 23, 53, 54]. Using the transmission electron microscopy technique, some authors have described a lower mitochondrial content in oocyte cytoplasm of endometriosis patients [5, 49]. Other authors, in cytogenetic studies performed using fluorescence in situ hybridization (FISH) on first polar bodies, have found abnormalities in the segregation of certain autosomes in patients with endometriosis [52].

Concerning our secondary objectives, the presence of deep pelvic endometriosis lesions or endometrioma(s) had no deleterious impact on either the global oocyte morphology or the success rates of IVF-ICSI attempts in the endometriosis group. These findings of no impact of the presence of endometrioma(s) on the results of IVF-ICSI are consistent with what has been previously published [27, 55–59]. Interestingly, analysis of the details of oocyte morphological abnormalities found significantly more misshapen oocytes in the subgroup of patients with endometrioma(s), without impacting the AOQI and MOMS scores (data presented in the supplemental table). Some authors suggest that the presence of endometrioma(s) may induce local pelvic inflammation and higher oxidative stress in the ovarian cortex, which may alter follicular and oocyte development and therefore potentially the oocyte morphology [60, 61].

The number of follicles over 15 mm, the number of retrieved oocytes and the number of mature oocytes were significantly lower in the endometriosis group, as previously described by other authors [5, 38]. Likewise, endometriosis group had significantly fewer embryos obtained, fewer good quality embryos and significantly lower cumulative clinical pregnancy, probably as a result of a significantly lower number of mature oocytes retrieved. Most of previous publications also report a lower number of mature oocytes and embryos in the case of endometriosis [5, 38, 59, 62]. Nevertheless, Juneau et al. [63], in a large retrospective study using the preimplantation genetic screening (PGS) technique, found similar embryonic aneuploidy rates between endometriosis and controls.

In our study, the lower cumulative clinical pregnancy and implantation rates in the endometriosis group may be explained more by reduced implantation capacity in patients with endometriosis than by impaired oocyte quality, as recently reported in several studies [64–66].

The statistical analysis of the outcomes of IVF-ICSI attempts according to the stimulation protocols in the endometriosis group seems to indicate a neutral impact of pre-treatment with GnRH agonists, combined oral or non-oral contraceptives or high dose of progestogens prior to the attempt on oocyte morphology. Moreover, the use of one of these 3 pre-treatments does not seem to have a negative impact on the cumulative clinical pregnancy rates. Other authors claimed a possible significant increase of pregnancy rates when using GnRH agonist pre-treatment or combined oral or non-oral contraceptives prior to IVF ± ICSI [59], as it was recently highlighted by De Ziegler et al. [67].

In our unit, we still use the policy of embryo transfer on day 2 or 3 because the evidence of an increasing of clinical or live pregnancy rate following fresh blastocyst transfer in comparison with a fresh cleavage stage transfer are low [68, 69]. Despite a recent study [70] suggesting better cumulative live birth change using blastocyst stage transfer strategy, there is to date no consensus about the first line transfer-policy due to conflicting results. Indeed, the cumulative pregnancy rate derived from fresh and frozen-thawed cycles following a single oocyte retrieval are usually similar between day 2 or day 3 transfer and blastocyst transfer [71].

The principal limitation of our study results from the retrospective design of the analysis. But the strength is that the analysis included more than 6,000 metaphase II oocytes, of which more than 2,000 were from endometriosis patients. Thus, in our knowledge, this is the largest study to have analyzed so precisely the morphology of mature oocytes using seven criteria and two scores in women with endometriosis undergoing IVF-ICSI. Moreover, because histologic or laparoscopic confirmation was not required to determine the presence or absence of endometriosis, some patients in the control group may in fact have a paucisymptomatic endometriosis that was not detected by US or MRI. Nevertheless, these procedures were performed by experienced clinicians in the detection of endometriosis lesions, as we manage a large population of women with this disease in our university hospital.

In the current study, endometriosis patients were significantly older, thinner and more often smokers than the control patients. Lower BMI in patients with endometriosis has already been reported in epidemiological studies [72, 73]. The association between smoking and endometriosis is controversial in the literature [73]. Furthermore, age difference between the two groups is probably related to the delay imposed by the diagnosis and exploration of endometriosis before IVF attempt. Nevertheless, from a clinical point of view, a difference of one year and/or 1.5 kg/m2 of BMI does not seem to have a significant impact in clinical practice. Similarly, the percentage of patients who smoked remains low in both groups. Moreover, the adjustment made to the statistical results took into account, among other factors, these 3 criteria, which allowed a reliable comparison of the two groups.

## Conclusion

In conclusion, endometriosis does not seem to have a negative impact on oocyte morphology in IVF-ICSI. Despite experimental data suggesting a decrease in intrinsic oocyte quality in patients with endometriosis, this cannot be unraveled by the study of oocyte morphology. The use of other sophisticated but invasive methods could be more appropriate to assess oocyte quality in women with endometriosis. Moreover, the development of other non-invasive methods that can be used routinely may provide additional information.

## Supplementary Information

**Additional file 1:**

## Data Availability

The datasets used and/or analysed during the current study are available from the corresponding author on reasonable request.

## Abbreviations

IVF: In Vitro Fertilization
ICSI: Intracytoplasmic Sperm Injection
AOQI: Average Oocyte Quality Index
MOMS: Metaphase II Oocyte Morphological Scoring System
BMI: Body Mass Index
MRI: Magnetic Resonance Imaging
CNGOF: French National College of Ob-Gyn
AMH: Anti-Müllerian Hormone
COH: Controlled Ovarian Hyperstimulation
FSH: Follicle Stimulating Hormone
HMG: Human Menopausal Gonadotropin
AFC: Antral Follicle Count
hCG: Human Chorionic Gonadotropin
GnRH: Gonadotropin Releasing Hormone
PB: Polar Body
PVS: Perivitelline Space
GEE: Generalized Estimating Equation
FISH: Fluorescence in situ hybridization
