# Supplementary material for: Impact of endometriosis on oocyte morphology in IVF-ICSI: retrospective study of a cohort of more than 6000 mature oocytes
Source: Reprod Biol Endocrinol. 2021 Oct 16;19:160. doi: 10.1186/s12958-021-00798-x (PMC8522159; doi:10.1186/s12958-021-00798-x)
Supplement: Supplementary file 1 — Additional file 1: [file 12958_2021_798_MOESM1_ESM.pdf]

Supplemental table: Comparison of oocyte morphology according to the characteristics of endometriosis: anomaly detail

	Endometrioma(s) $n_{ovo\ M2} = 889$	No Endometrioma(s) $n_{ovo\ M2} = 1087$	*p*	Deep Pelvic Endometriosis $n_{ovo\ M2} = 986$	No Deep Pelvic Endometriosis $n_{ovo\ M2} = 905$	*p*	Endometriosis Surgery $n_{ovo\ M2} = 1294$	No Endometriosis Surgery $n_{ovo\ M2} = 674$	*p*
Fragmented PB1	463 (52.1)	595 (54.7)	0.32	534 (54.2)	471 (52.0)	0.85	689 (53.2)	374 (55,5)	0.65
Abnormal ZP (irregular shape or thickened)	22 (2.5)	24 (2.2)	0.43	37 (3.8)	28 (3.1)	0.83	45 (3.5)	20 (3,0)	0.59
Large PVS	136 (15.3)	134 (12.3)	0.32	139 (14.1)	118 (13.0)	0.46	167 (12.9)	98 (14,5)	0.42
Peri-Vitelline material	170 (19.1)	197 (18.1)	0.31	178 (19.7)	169 (17.1)	0.65	238 (18.4)	140 (20,8)	0.12
Abnormal oocyte shape	68 (7.6)	53 (4.9)	**0.018**	66 (6.7)	45 (5.0)	0.19	82 (6.3)	36 (5,3)	0.48
Granular cytoplasm	140 (15.7)	139 (12.8)	1.00	147 (14.9)	131 (14.5)	0.59	181 (14.0)	107 (15,9)	0.84
Intracytoplasmic vacuoles	22 (2.5)	24 (2.2)	0.61	20 (2.0)	24 (2.7)	0.34	30 (2.3)	16 (2,4)	0.97

Values are presented by number of oocytes and percentage, unless otherwise stated. No missing data for these parameters.

PB1: First Polar Body ; ZP: zona pellucida ; PVS: Perivitelline Space
